# A Protective and Pathogenic Role for Complement During Acute *Toxoplasma gondii* Infection

**DOI:** 10.3389/fcimb.2021.634610

**Published:** 2021-02-22

**Authors:** Patricia M. Sikorski, Alessandra G. Commodaro, Michael E. Grigg

**Affiliations:** ^1^Molecular Parasitology Section, Laboratory of Parasitic Diseases, National Institute of Allergy and Infectious Diseases, National Institutes of Health, Bethesda, MD, United States; ^2^Department of Microbiology and Immunology, Georgetown University Medical Center, Georgetown University, Washington, DC, United States

**Keywords:** complement, *Toxoplasma gondii*, C4BP, factor H, regulation, immune evasion

## Abstract

The infection competence of the protozoan pathogen *Toxoplasma gondii* is critically dependent on the parasite’s ability to inactivate the host complement system. *Toxoplasma* actively resists complement-mediated killing in non-immune serum by recruiting host-derived complement regulatory proteins C4BP and Factor H (FH) to the parasite surface to inactivate surface-bound C3 and limit formation of the C5b-9 membrane attack complex (MAC). While decreased complement activation on the parasite surface certainly protects *Toxoplasma* from immediate lysis, the biological effector functions of C3 split products C3b and C3a are maintained, which includes opsonization of the parasite for phagocytosis and potent immunomodulatory effects that promote pro-inflammatory responses and alters mucosal defenses during infection, respectively. In this review, we discuss how complement regulation by *Toxoplasma* controls parasite burden systemically but drives exacerbated immune responses locally in the gut of genetically susceptible C57BL/6J mice. In effect, *Toxoplasma* has evolved to strike a balance with the complement system, by inactivating complement to protect the parasite from immediate serum killing, it generates sufficient C3 catabolites that signal through their cognate receptors to stimulate protective immunity. This regulation ultimately controls tachyzoite proliferation and promotes host survival, parasite persistence, and transmissibility to new hosts.

## Introduction

Insect and vertebrate complement systems play critical roles in the defense against invading microbial pathogens and the regulation of inflammatory responses. Apicomplexan parasites, which comprise a diverse group of obligatory intracellular parasites, have evolved sophisticated strategies to regulate or inactivate this humoral first line of defense to promote their infection competency in both insects and mammalian hosts (Belachew, 2018; Shao et al., 2019). These parasites possess complex lifecycles consisting of both sexual and asexual stages that typically infect multiple hosts, and so, must overcome a myriad of species-specific immune defenses in order to initiate infection. The most studied among these parasites include *Toxoplasma*, *Cryptosporidium*, *Eimeria*, and *Plasmodium*, which cause various infectious diseases of human and veterinary importance (Votýpka et al., 2017). This paper reviews the strategies employed by *Toxoplasma gondii* to both inactivate and regulate the complement cascade and highlights similar strategies employed by other Apicomplexan parasites to facilitate the establishment of a persistent, transmissible infection.

## The Complement System

**Figure 1:**
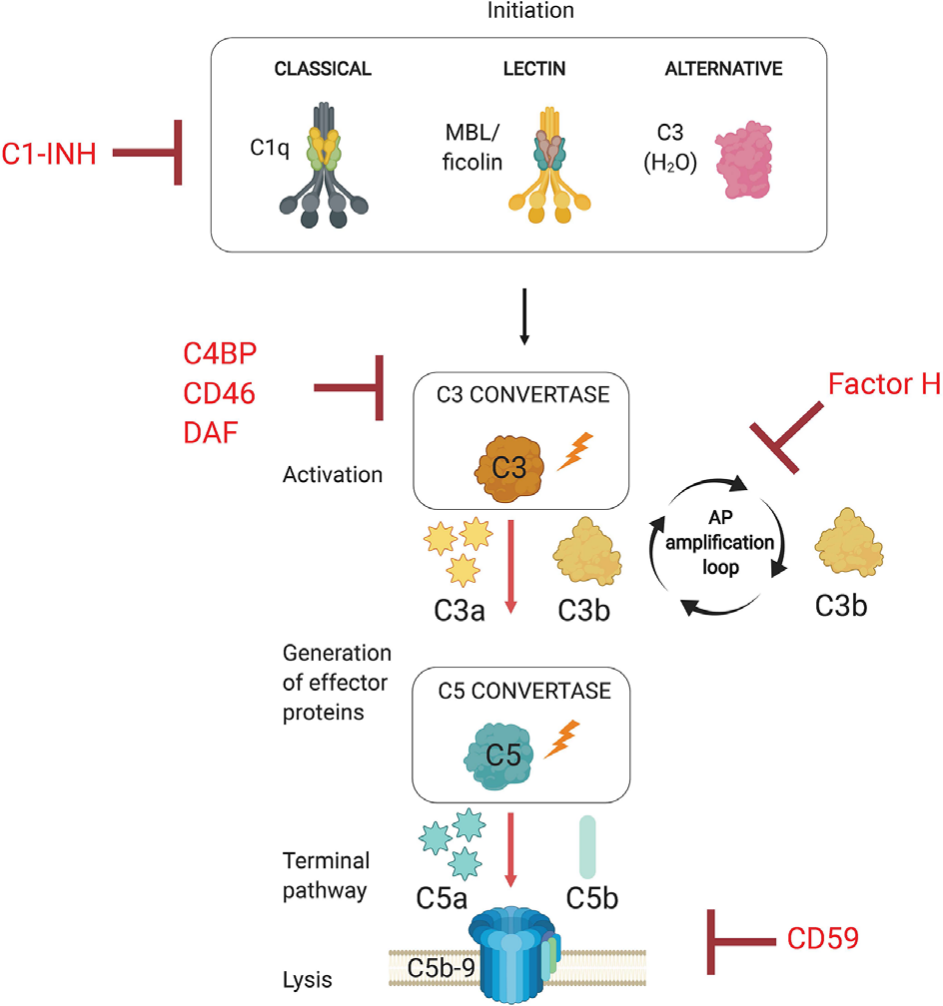
Overview of the Complement System. The complement system is activated *via* three separate pathways (classical, lectin, and alternative) that have distinct recognition mechanisms (C1q, MBL/ficolins, and C3b(H_2_O), respectively). Complement activation results in the formation of C3 convertases. All three pathways converge at C3 activation *via* C3 convertases, which generates effector molecules C3a and C3b. C3b allows for progression to the terminal pathway by forming C5 convertases, which cleave C5 into C5a and C5b. The terminal pathway requires C5 activation product C5b to initiate the assembly of the membrane attack complex (MAC, C5b-9). The pore inserts into the cell membrane and promotes pathogen lysis. Complement regulators target complement pathway initiation (C1-INH, C1 esterase inhibitor), convertase formation (C4BP, C4b-binding protein; CD46; DAF, decay accelerating factor), and the alternative pathway amplification loop (Factor H).

The complement system is an evolutionarily conserved first line of defense that rapidly activates against invading pathogens. This defense system consists of a set of circulating liver-derived soluble proteins, membrane bound receptors, and regulators that function in a highly coordinated proteolytic cascade to opsonize and lyse invading microbes in addition to mobilizing the cellular arm of the immune response (**Figure 1**—schematic of complement system pathway activation and regulation). Activation of this cascade of more than 50 molecules and their complement receptors occur *via* three different pathways: the classical (CP), lectin (LP), and alternative (AP) pathways. Activation of these pathways leads to the formation of pathway-specific complexes known as C3 convertases, in which all three pathways converge to facilitate the cleavage of the central molecule C3 into effector proteins C3a and C3b. Successful complement activation culminates in the assembly of a pore-forming protein on the pathogen surface (referred to as the membrane attack complex, or MAC) that mediates pathogen lysis.

The complement system is activated upon detection of invading pathogens through specialized and pathway-specific recognition molecules that recognize pathogen-associated molecular patterns (PAMPs). Initiation of the classical pathway requires the recognition of pathogen bound IgM or IgG by pattern recognition molecule C1q. The lectin pathway is activated when microbial sugars are bound by the pattern recognition molecule mannose-binding lectin (MBL). The alternative pathway, however, does not rely on a recognition molecule for activation, but rather on the failure to regulate the continuous low-level spontaneous hydrolysis of C3 into C3(H_2_O), which indiscriminately probes foreign and self surfaces alike. The ability of the AP to discriminate between self and non-self surfaces relies on the presence of membrane bound and soluble complement regulatory proteins. Importantly, the AP is a critical component of host defense because it amplifies the complement response independent of the pathway that initiates the response, so it represents a critical target for regulation by host and pathogens alike. Specifically, the AP utilizes CP and LP activated C3b as a platform for rapidly generating new AP C3 convertases which establishes a positive feedback loop to amplify C3 cleavage (Thurman and Holers, 2006). Due to its non-discriminatory nature and potential for rapid amplification, this process must necessarily be tightly controlled by host regulator proteins to limit inflammation and damage to host cells. Complement regulators target various points within the proteolytic cascade, which includes inactivation of proteases associated with C1q and mannose binding lectin (MBL), cleavage of active C3b into inactive iC3b and C3dg, accelerating the decay of C3 and C5 convertases, and preventing insertion of the membrane attack complex into the plasma membrane (Schmidt et al., 2016).

**Figure 2:**
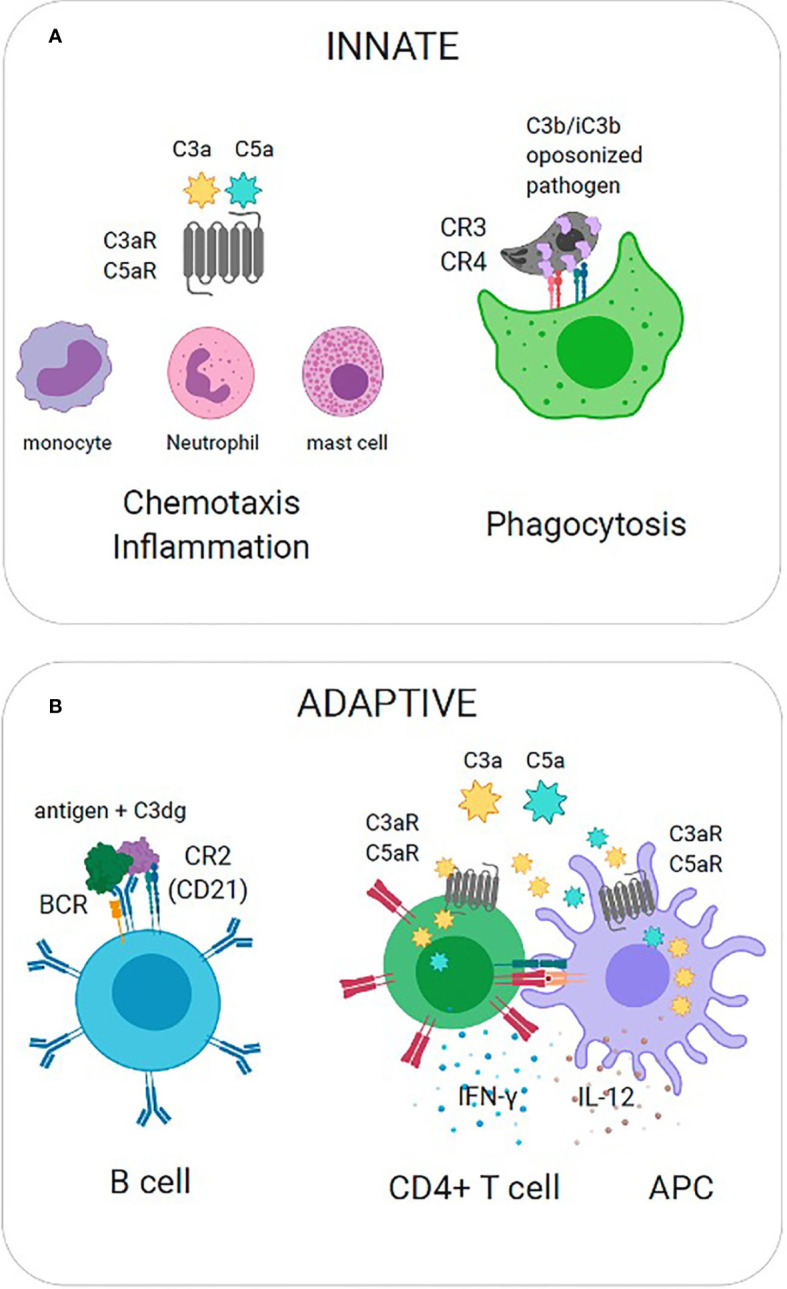
Effector Functions of Complement Split Products. **(A)** Innate immunity. (Left) C3a is recognized by cognate receptor C3aR to mediate recruitment of innate immune cells (mast cells, monocytes, neutrophils) and promote inflammation. (Right) C3b is covalently coupled to pathogen surfaces and promotes opsonization. C3b is recognized by complement receptors (CR3, CR4) to promote phagocytosis. **(B)** Adaptive immunity. (Left) Co-ligation of complement receptor 2 with the B cell receptor (BCR) on B cells *via* C3dg-antigen complexes amplifies B cell signaling and lowers the threshold for B cell activation. (Right) The local activation and secretion of complement anaphylatoxins (C3a, C5a) and upregulation of cognate receptors (C3aR, C5aR) is triggered during T cell-APC interactions. Autocrine and paracrine signaling through C3aR and C5aR promote Th1 cytokine production (IL-12, IFN).

Protection against pathogens occurs when the complement system produces several biologically active effector molecules, which include opsonins (C3b and its catabolites iC3b, C3d and C3dg), anaphylatoxins (C3a and C5a), and the membrane attack complex (MAC). The most direct effector function is the lysis of pathogens through MAC. Because many pathogens have evolved mechanisms to limit complement activation and prevent the progression of the cascade, generation of C3b and anaphylatoxins ensure additional layers of immune defense. These effector functions are exerted by the interaction between complement split product effectors and their host receptors to facilitate biological processes such as phagocytosis, chemotaxis, and inflammation (**Figure 2A****)**. In the absence of antibodies, C3b and its catabolites (iC3b, C3d, and C3dg) function as major opsonins recognized by complement receptors 1, 3, and 4 (CR1, CR3, CR4) that are expressed on myeloid cells and aid in phagocytosis and pathogen clearance. Anaphylatoxins C3a and C5a are critical danger signals that exert their function through interactions with cognate receptors C3aR and C5aR expressed on both immune and non-immune cells. Signaling through these receptors induces several critical pro-inflammatory immunological responses, including chemotaxis, oxidative burst, immune cell activation, vasodilation and induction of cytokines (Klos et al., 2009). Complement split products also play a critical role in bridging innate immunity and adaptive immunity (**Figure 2B**). For example, C3b inactivation products C3dg and C3d covalently linked to antigen are recognized by complement receptor 2 (CR2, CD21) expressed on B cells. Co-ligation of CR2 with the B cell receptor (BCR) *via* C3d-antigen complexes amplifies B cell signaling, lowers the threshold for B cell activation, and thus this interaction is critical for enhancing B cell responses (Carroll, 2008). Recent work has also implicated local intracellular complement activation and the subsequent production of anaphylatoxins (C3a, C5a) during cognate interactions between antigen presenting cells (APCs) and CD4+ T cells (Heeger et al., 2005; Strainic et al., 2008). Upregulation of and signaling through cognate receptors C3aR and C5aR have been shown to regulate T cell activation, lineage commitment and proinflammatory Th1 cytokine production (Strainic et al., 2008; Liszewski et al., 2013).

## Complement Activation and Resistance: The Apicomplexa

Apicomplexan parasites, not unlike bacteria, viruses, and fungi, activate the complement system. Complement evasion is a critical step in the establishment of infection, hence parasites have evolved multiple sophisticated strategies to overcome serum killing. Mechanisms of parasite complement evasion include the recruitment of host regulators by parasite surface molecules, expression of complement regulator protein orthologs, and expression of parasite-encoded proteins that target and/or inactivate complement function (recently reviewed by Shao et al., 2019).

While parasites share common evasion strategies, these mechanisms are achieved by unique parasite-specific factors. *Plasmodium* spp. evades both mosquito and human complement systems to facilitate the survival and transmission of the parasite from vector to host. Genetic studies have identified the 6-CYS protein Pfs47 as a critical factor facilitating parasite transmission in mosquitos by its ability to regulate the insect complement-like immune system (Molina-Cruz et al., 2013). Additional recently identified parasite factors that recruit human regulator Factor H to facilitate C3b inactivation include pfGAP50 expressed by gametes in the mosquito midgut and Pf92 expressed during the blood-stage (Simon et al., 2013; Kennedy et al., 2016). In addition, parasites bind plasminogen during the intraerythrocytic stage and mediate its conversion to plasmin in order to inactivate C3b, however the parasite factor(s) that facilitate this interaction have not been determined (Reiss et al., 2021).

Complement evasion by *Cryptosporidium* and *Toxoplasma*, on the contrary, is less extensively studied. While studies have established that *Cryptosporidium parvum* binds C3 and classical (C1q) and lectin (mannose-binding lectin, MBL) pathway molecules (Petry et al., 2008), little is known about resistance mechanisms. Our group has confirmed earlier work demonstrating that *Toxoplasma* inactivates C3 (Fuhrman and Joiner, 1989; Sikorski et al., 2020) but the precise molecular details have only recently been elucidated. We utilized improved strategies to study complement interactions with *T. gondii* using flow cytometry, leading to a significant advancement in our understanding of complement activation and regulation by *T. gondii*. Our work has for the first time revealed important contributions of the lectin pathway as well as parasite genotype to complement activation. Our studies showed strain-specific differences in C3b deposition between Type I and Type II strains that was partially attributed to the differences in surface glycans and lectin pathway activation, indicating that increased C3b deposition on Type II strains correlated with greater lectin binding and MBL recognition. However, despite these differences, both strains were equally capable of inactivating C3b and were resistant to serum killing. Our data showed that resistance to serum killing was due to the ability of *T. gondii* to recruit the alternative pathway regulator Factor H (FH) and classical and lectin pathway regulator C4b-binding protein (C4BP) to the parasite cell surface (Fuhrman and Joiner, 1989; Sikorski et al., 2020). The importance of the alternative pathway was highlighted by its critical role mediating serum resistance, irrespective of its limited contribution in the initiation of the complement system. Our studies demonstrated that blocking Factor H, but not C4BP, resulted in greater C5b-9 deposition and greater parasite susceptibility to serum killing, suggesting that the AP functions principally as a potent mediator that amplifies complement activation and is thus an important target for *T. gondii* regulation. Although the *T. gondii* parasite factor(s) that bind C3b, FH, and C4BP have not been identified, their discovery would directly address the role of individual and cumulative parasite factors in the molecular basis of protection against serum killing, and would further provide an opportunity to unearth potential targets for therapeutic intervention to render parasites more susceptible to complement attack. Collectively, these studies demonstrate how apicomplexan parasites employ multiple strategies to target C3 in order to successfully resist serum killing.

## Complement: Protective and Pathological Role During *T. gondii* Infection

*Toxoplasma gondii* is a highly prevalent and successful protozoan parasite that can infect any nucleated cell in all mammals. Non felid, intermediate hosts acquire *T. gondii* by ingesting oocysts from contaminated water or food, or by eating infected meat containing tissue cysts. Upon ingestion, parasites excyst and invade the intestinal epithelium where they differentiate and rapidly replicate asexually as tachyzoites. The parasites then disseminate systemically to distal organs to mediate acute infection before establishing a chronic infection by the formation of tissue cysts (Dubey et al., 1997). The pathogenic tachyzoite form can be easily grown *in vitro* and chronic stages can be maintained in animal models, thus mice represent an experimentally tractable model system optimal for studying this host-pathogen interaction.

Until recently, *T. gondii* complement resistance mechanisms and the biological significance of the complement system *in vivo* during acute infection were unknown. The role of complement during systemic *T. gondii* intraperitoneal infection was recently evaluated using complement deficient C57BL/6 mice. In the absence of C3, mice died acutely and exhibited higher parasite loads whereas control mice survived. This study demonstrated that both the presence of C3 as well as the parasite’s ability to inactivate C3 are important factors regulating parasite proliferation and contributing to host survival *in vivo* (Sikorski et al., 2020). One limitation of using C3 deficient animals is that the protective effect observed could not be specifically assigned to either C3a or C3b split product effector function (Sikorski et al., 2020). The observed reduction in *T. gondii*-specific antibodies in this model was, however, consistent with the known function of C3dg tagged antigen to enhance humoral responses (Sikorski et al., 2020), suggesting that C3b opsonization of *T. gondii* antigen is critical for priming humoral immunity. Importantly, this study suggested that *T. gondii* strikes a critical balance in fine tuning complement system activation, by evading serum killing to promote parasite persistence, while preserving the ability of complement effectors C3a and C3b to activate sufficient host immunity to regulate parasite proliferation and promote both host survival and parasite transmissibility.

Other studies have attempted to elucidate the role of anaphylatoxin signaling in acute *T. gondii* infection. Anaphylatoxin signaling has been implicated in regulating Th1 responses, which are critical in host resistance to *T. gondii* infection. The first studies demonstrated increased susceptibility of *C5ar1*^−/−^ *C3ar1*^−/−^ double knockout (DKO) mice to *T. gondii* infection. Intraperitoneal injection of 20 Me49 cysts resulted in acute death within 12 days in the DKO mice whereas all WT mice survived for greater than 50 days, the longest time point studied. The DKO mice had significant reductions in IL-12 and IFN-γ secretion in splenic cultures stimulated with STAg (soluble *Toxoplasma* antigen) and supported a protective role for anaphylatoxin receptor signaling (Strainic et al., 2008). These findings are consistent with an established role for locally produced C3a and C5a in regulating Th1 responses through autocrine and paracrine C3aR/C5aR receptor signaling (Strainic et al., 2008; Liszewski et al., 2013). More recently, infection studies in *C5ar1*^−/−^ mice showed that these mice also die acutely, but with a much less dramatic phenotype or kinetic than the DKO mice (Briukhovetska et al., 2020). In this study, 50 cysts of the Type II strain Me49 were injected intraperitoneally. At this dose, both WT and *C5ar1*^−/−^ mice were susceptible to intraperitoneal infection, with 60% of WT mice dead within 30 days compared to 80% of *C5ar1*^−/−^ infected mice, which exhibited a statistically significant higher parasite load during acute disease (Briukhovetska et al., 2020). The authors argued that C3b and its degradation products likely promoted the release of the anaphylatoxin C5a that bound the C5aR1 expressed on CD8α+ dendritic cells (DCs) which were activated by *T. gondii*-induced TLR signaling to amplify IL-12 and IFN-γ production (Briukhovetska et al., 2020). Evidence for decreased levels of serum proinflammatory cytokines and increased IL-10 serum levels in *C5ar1*^−/−^ mice, and the reduction of IL-12 secretion from *C5ar1*^−/−^ splenic DCs in response to STAg *in vitro* suggested that complement signaling was important for priming appropriate Th1 responses to control parasite replication. Collectively, these studies suggest that loss of systemic complement and anaphylatoxin receptor signaling play a significant role in the failure to sense or initiate proper humoral and cytokine responses against acute *T. gondii* infection, respectively, which is necessary to control parasite proliferation during acute disease.

To better understand the relative and contributing roles of complement effectors C3, C3b, and associated anaphylatoxin receptor signaling on immunological priming and control of parasite proliferation, we infected C57BL/6J mice perorally with 40 cysts of the Me49 strain. C57BL/6 mice develop an acute, Th1 CD4+ T cell-mediated lethal ileitis within 8 days of *Toxoplasma* peroral infection that results in tissue destruction and necrosis of the intestinal mucosa. This immunopathology is associated with exacerbated Th1 immune responses, which include increased production of the inflammatory mediators IFNγ, TNF-α, nitric oxide (Liesenfeld et al., 1996; Khan et al., 1997; Liesenfeld et al., 1999), Th1 and Th17 cytokines (Rachinel et al., 2004; Vossenkämper et al., 2004), and shifts in the intestinal microbiota (Heimesaat et al., 2006; Molloy et al., 2013; Wang et al., 2019). Systemic complement and anaphylatoxin receptor signaling is emerging as an important component of the intestinal immune response by its ability to regulate epithelial barrier integrity, the microbiota, and oral tolerance (Pekkarinen et al., 2013; Nissilä et al., 2017; Zhang et al., 2018; Benis et al., 2019; Qi et al., 2020), however the contribution of complement in the protection of mucosal barriers during acute *T. gondii* infection has not been directly addressed.

**Figure 3:**
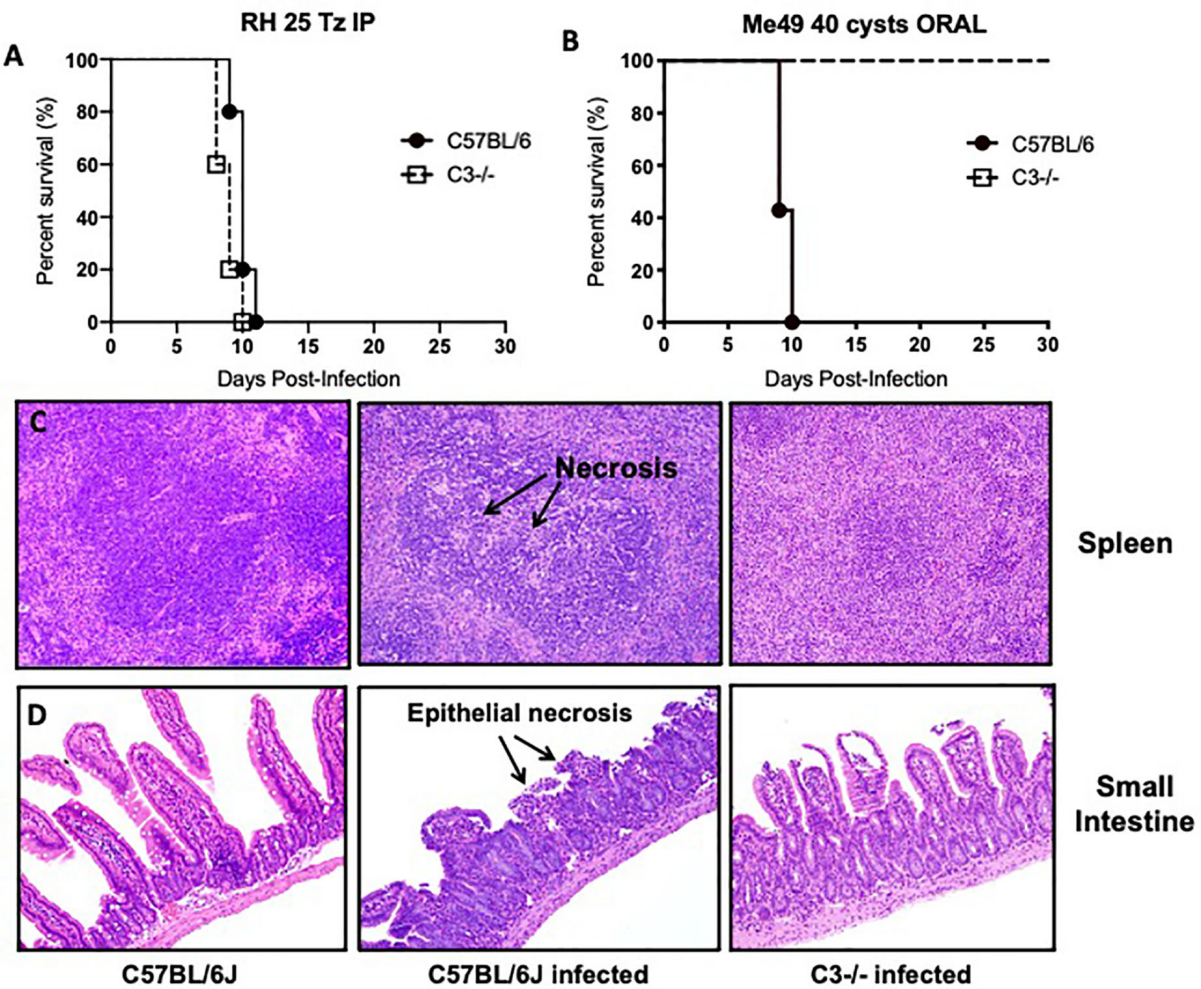
Lethal ileitis in *Toxoplasma gondii* perorally infected C57BL/6J mice is dependent on C3. **(A)** Survival of 6- to 8-week-old C57BL6/J (n = 5, closed circle) and C3^−/−^ (n=5, open square) female mice infected with 25 tachyzoites injected intraperitoneally of the RH strain of *Toxoplasma gondii*. Data shown are for one of two independently performed experiments. **(B)** Survival of 6- to 8-week-old C57BL6/J (n=5, closed circle) and C3^−/−^ (n=5, open square) female mice infected by oral gavage with 40 cysts of the Me49 strain of *Toxoplasma gondii*. Data shown are for one of two independently performed experiments. **(C)** Histological examination of the spleen of C57BL6/J or C3^−/−^ mice stained with hematoxylin-eosin (HE) at day 8 post-infection with 40 cysts of Me49 by oral gavage. **(D)** Histological examination of the small intestine of C57BL6/J or C3^−/−^ mice stained with hematoxylin-eosin (HE) at day 8 post-infection with 40 cysts of Me49 by oral gavage.

To investigate this question, we infected C57BL/6J WT and C3 deficient animals with 40 Me49 tissue cysts perorally and assessed survival. As a control, we also infected WT and C3 deficient animals with 25 tachyzoites of the Type I RH strain intraperitoneally to demonstrate that C3 is protective during infection regardless of parasite genotype (**Figure 3A**). In stark contrast to the i.p. model in which all mice die acutely, 100% of C3 deficient mice survived *T. gondii* infection whereas all WT mice died acutely, within 10 days (**Figure 3B****)**. Histologic examination of the spleen and intestines at days 4, 6, and 8 (**Figures 3C, D****)** post infection showed striking differences in pathology, highlighted by areas of necrosis in the white pulp of the spleen of infected WT mice compared to C3^−/−^ mice, and a severe necrosis of the ilea, predominantly within the villi, with significant inflammation, a loss of columnar epithelial cells and a large accumulation of granulocytes that was largely absent in the C3^−/−^ infected mice. Parasite load was determined by plaquing a portion of the spleen and small intestine of WT versus C3 deficient mice at day 6 post infection and showed a similar level of parasites present (with no significant difference detected in the tissues examined; data not shown).

Consistent with phenotypes observed in other murine models of inflammatory-driven acute colitis, complement deficient mice were protected from *T. gondii*-induced lethal ileitis and survived. We hypothesize that dysregulation of the local complement response contributed detrimental inflammation and led to the pathogenesis of acute infection. Similar phenotypes have previously been observed in perorally infected mice deficient in the inducible NO synthetase enzyme (iNOS^−/−^) and may suggest that complement likewise plays an analogous role by exacerbating the immunopathology observed in this oral model of acute ileitis. Nitric oxide is an important mediator restricting intracellular pathogen growth. iNOS^−/−^ mice exhibited greater dissemination and parasite burden but survived significantly longer than control mice infected perorally (Khan et al., 1997; Scharton-Kersten et al., 1997). Whereas control mice exhibited exacerbated cytokine production and necrosis, the prolonged survival of iNOS^−/−^ mice was attributed to control of the dysregulated inflammatory response that occurs in B6 mice. Unlike the C3 deficient mice, iNOS^−/−^ mice eventually succumb to infection, largely the result of their inability to control parasite proliferation (Khan et al., 1997). These studies indicated that iNOS and NO production is critical for parasite killing. Further, these findings suggest that acute ileitis is critically dependent on the level of IL-10 present, a critical meditator of immune homeostasis during proinflammatory Th1 responses, both in genetically susceptible C57BL/6 mice and resistant BALB/c. Indeed, mice deficient in IL-10 show an increased susceptibility to *T. gondii* infection compared to control animals (Gazzinelli et al., 1996; Suzuki et al., 2000). The increased susceptibility was not attributed to increases in parasite burden, but rather to the inability to control the pro-inflammatory response. Together, the studies highlighted illustrate that several immunological factors contribute toward tipping the balance towards immunopathology in *T. gondii*-induced ileitis, and further investigations are required to determine how protective versus pathological roles of complement contribute to the immunopathology associated with oral infection of genetically susceptible C57BL/6 mice. In the next section, we discuss how studies addressing the role of complement in other intestinal inflammatory disorders may provide insight into the observed dichotomous role for complement in the pathogenesis of acute *T. gondii* peroral infection.

## Additional Insights and Outstanding Questions

The intestinal pathology induced by oral *T. gondii* infection shares similarities with human inflammatory bowel disease (IBD) (Liesenfeld, 2002). Recent studies in the IBD field support a model in which complement has both a pathogenic and a protective role and specifically, that local complement production plays a central role in the pathophysiology of these inflammatory diseases, including the established experimental mouse model of acute dextran-sulfate induced (DSS) colitis, a model for human inflammatory bowel disease. Complement is also produced extra-hepatically by immune cells and epithelial cells, including intestinal enterocytes, and may have specific functions at local sites (Morgan and Gasque, 1997; Lubbers et al., 2017). Mice deficient in C3 or Factor B were protected from acute colitis induction 5 days post DSS treatment and exhibited improved clinical outcome (Elvington et al., 2015). In contrast to previous studies (Lu et al., 2010), complement deficient mice unexpectedly died within 5 days of the DSS recovery period, indicating a protective role for complement after induction of colitis (Schepp-Berglind et al., 2012). Mortality was attributed to impaired epithelial barrier function in the absence of complement, leading to translocation of commensals and increased endotoxins, and reduction of mucosal tissue proliferation during the repair process (Schepp-Berglind et al., 2012; Elvington et al., 2015). C5 deficiency in an earlier study was also shown to make mice more susceptible to colitis after 10 days, supporting this protective role (Deguchi et al., 2005). Reported discrepancies in phenotypes across various studies are likely due to differences in disease severity attributed to dose of DSS and mouse genotype. Thus, understanding the contributions of complement to dual protective and pathogenic functions in *T. gondii* infection may not only rely on host genotypes but also parasite genotype and dose and thus requires further study.

Anaphylatoxin receptor signaling has also been shown to play a role in regulating pro-inflammatory responses at the intestinal barrier. Reports of a pathogenic role for C5a in colitis models are supported by the amelioration of disease pathology in mice and rats using C5aR deficient animals (Johswich et al., 2009), C5a blockade (Chen et al., 2011) or treatment with C5a agonists (Woodruff et al., 2003). Complement activation also contributed to the development of colitis-associated colorectal cancer (CAC), supported by tumor repression in complement deficient mice (C3, C5, or C5aR) (Ning et al., 2015). Mechanistic studies revealed that complement deficiency reduced proinflammatory cytokine production produced by neutrophils (IL-1*β* and IL-17) in the colonic tissues, indicating that C5a is a potent inducer of this response. While C3aR deficiency in BALB/c was partially protective in DDS-induced colitis, there was no significant effect observed in C57BL/6 mice (Wende et al., 2013), indicating that differences between these two mice strains are important factors in these phenotypes. Given this evidence and the recently demonstrated role of C5aR signaling in systemic *T. gondii* infection (Briukhovetska et al., 2020), it is highly likely that anaphylatoxins play an important role in promoting inflammation in the gut during *T. gondii* infection.

It is tempting to speculate that complement at mucosal barriers may need greater regulation. IBD in humans and mice is associated with increased complement activation (Ahrenstedt et al., 1990; Elvington et al., 2015; Preisker et al., 2019) and reduced expression of complement regulatory proteins in the gut epithelium (Berstad and Brandtzaeg, 1998; Scheinin et al., 1999). Accordingly, mice deficient in DAF (CD59) are more susceptible to DSS-induced colitis (Lin et al., 2004). In the previously discussed studies, while complement deficient mice died by day 5 after DSS recovery, the wild type mice treated with complement inhibitors survived the DSS treatment, suggesting complement regulation or inhibition over deficiency is more protective and may have important therapeutic implications for inflammatory conditions (Schepp-Berglind et al., 2012; Elvington et al., 2015).

Lastly, the interaction between intestinal complement, commensal bacteria, and *Toxoplasma* during acute infection remains to be fully elucidated. *T. gondii* infection is associated with microbial dysbiosis (Molloy et al., 2013; Wang et al., 2019) and emerging evidence for the role complement plays in maintaining gut homeostasis and regulating commensal microbiota is a factor that must be carefully evaluated (Benis et al., 2019; Qi et al., 2020). Equally important is to consider whether the interaction of *T. gondii* with complement and its regulators may contribute adversely to the outcome of this complex environment. Our previous work has shown that parasite genotype impacts complement deposition. Additional studies are required to test if the capacity of Type II strains, that show greater C3b deposition (Sikorski et al., 2020) could potentially induce greater local levels of C3a and C5a, which play a role in pro-inflammatory signaling by their cognate anaphylatoxin receptors. It is also possible that greater opsonization of Type II strains may contribute to the increased internalization of Type II strains *via* phagocytosis. Recent studies have shown that avirulent Type II strains preferentially enter macrophages through phagocytosis but avoid elimination by escaping the phagolysosome in murine macrophages (Zhao et al., 2014), in which the authors hypothesized this mode of entry promotes an enhanced immune stimulation and greater control of acute infection.

The identification of parasite factors that activate and regulate complement are critical for determining whether direct parasite activation and regulation of the complement system is occurring *in vivo*. Studies from the *Plasmodium* field have identified several developmentally regulated 6-CYS surface proteins that regulate complement in both the mosquito and human host (Molina-Cruz et al., 2013; Kennedy et al., 2016). Comparative modeling studies and the crystal structure of *Pf12* has determined that the 6-CYS proteins share tertiary structural homology with *T. gondii* SRS (SAG-1 related sequences) surface proteins (He et al., 2002; Gerloff et al., 2005; Arredondo et al., 2012; Tonkin et al., 2013). This evidence points to a potential role for the structurally homologous *T. gondii* SRS surface proteins (Gerloff et al., 2005; Tonkin et al., 2013) to possess an analogous role. Though the mechanism for Factor H recruitment by the *Plasmodium* 6-CYS protein *Pf92* remains elusive, it is well established that FH is recruited to host cell surfaces through its ability to bind host ligands such as sialic acid and sulfated proteoglycans (SPGs) (Blaum, 2017). *Toxoplasma* is known to interact with both sialic acid and SPGs *via* microneme proteins 1 and 4 (MIC1, MIC4) and SRS57, and thus these proteins are prime candidates for future study to determine their capacity in recruiting FH (Ortega-Barria and Boothroyd, 1999; Dzierszinski et al., 2000; Jacquet et al., 2001; Sardinha-Silva et al., 2019). Interestingly, recent analyses done in our lab have determined that the developmentally regulated SRS superfamily of surface proteins are significantly expanded in *T. gondii* (Jung et al., 2004; Wasmuth et al., 2012). While the biological significance of this expansion remains elusive, it is tempting to speculate that the SRS superfamily plays a role in overcoming immunological barriers to establish successful infection in a wide host range. Specifically, are there stage-specific surface or secreted proteins that activate or regulate complement in a species-specific manner, *i.e.* intermediate versus definitive host, or does a universally expressed SRS protein facilitate this process across several species? All of these intriguing questions require additional studies.

## Concluding Remarks

The data presented in this review highlight a protective and pathogenic role for the complement system during acute *T. gondii* infection, depending on the route of infection. We are just beginning to understand how parasite modulation of complement activation may impact complement effector functions required to strike the optimal balance in the intermediate host. Evading serum killing ensures parasite survival, persistence and transmission to new hosts, while maintaining the generation of critical effector proteins (iC3b, C3a, C5a) that are required for stimulating sufficient humoral and Th1 immunity to regulate tachyzoite proliferation. We argue that future investigations will further unravel the immunomodulatory role of complement activation and regulation during *T. gondii* infection in the gut. Additional studies into the parasite factors that interact with and regulate complement are required to better understand the dynamics that occur between parasite and host systemically and locally in the gut.

## Author Contributions

Review topic was solicited by MG, PS, and AC. PS wrote the first draft with editing from MG and AC. All authors contributed to the article and approved the submitted version.

## Funding

This work was supported by the Intramural Research Program of the National Institute of Allergy and Infectious Diseases (NIAID) at the National Institutes of Health.

## Conflict of Interest

The authors declare that the research was conducted in the absence of any commercial or financial relationships that could be construed as a potential conflict of interest.
